# All-Fiber LITES Sensor Based on Hollow-Core Anti-Resonant Fiber and Self-Designed Low-Frequency Quartz Tuning Fork

**DOI:** 10.3390/s25092933

**Published:** 2025-05-06

**Authors:** Xiaorong Sun, Weipeng Chen, Ying He, Haiyue Sun, Shunda Qiao, Yufei Ma

**Affiliations:** 1 National Key Laboratory of Laser Spatial Information, Harbin Institute of Technology, Harbin 150001, China; sunxiaorong020228@163.com (X.S.); 15373356845@163.com (W.C.); sunhaiyue282@163.com (H.S.); shundaqiao@126.com (S.Q.); 2 Zhengzhou Research Institute, Harbin Institute of Technology, Zhengzhou 450008, China

**Keywords:** all-fiber, light-induced thermoelastic spectroscopy (LITES), hollow-core anti-resonant fiber (HC-ARF), self-designed low-frequency quartz tuning fork (QTF), sensor

## Abstract

In this paper, an all-fiber light-induced thermoelastic spectroscopy (LITES) sensor based on hollow-core anti-resonant fiber (HC-ARF) and self-designed low-frequency quartz tuning fork (QTF) is reported for the first time. By utilizing HC-ARF as both the transmission medium and gas chamber, the laser tail fiber was spatially coupled with the HC-ARF, and the end of the HC-ARF was directly guided onto the QTF surface, resulting in an all-fiber structure. This design eliminated the need for lens combinations, thereby enhancing system stability and reducing cost and size. Additionally, a self-designed rectangular-tip QTF with a low resonant frequency of 8.69 kHz was employed to improve the sensor’s detection performance. Acetylene (C_2_H_2_), with an absorption line at 6534.37 cm^−1^ (1.53 μm), was chosen as the target gas. Experimental results clearly demonstrated that the detection performance of the rectangular-tip QTF system was 2.9-fold higher than that of a standard commercial QTF system. Moreover, it exhibited an outstanding linear response to varying C_2_H_2_ concentrations, indicating its high sensitivity and reliability in detecting C_2_H_2_. The Allan deviation analysis was used to assess the system’s stability, and the results indicated that the system exhibits excellent long-term stability.

## 1. Introduction

With the accelerating process of industrialization, environmental issues have increasingly become a global focal point. In this context, the impact of trace gases cannot be overlooked. These gases, including but not limited to methane (CH_4_), carbon dioxide (CO_2_), carbon monoxide (CO), and acetylene (C_2_H_2_), not only play a pivotal role in industrial production but also contribute to the greenhouse effect, climate change, and safety risks. Therefore, the detection and quantification of trace gases have become crucial, as they are not only related to environmental protection but also to human health and industrial safety [1,2,3,4,5,6,7,8,9].

In the field of trace gas detection, laser absorption spectroscopy (LAS) technology distinguishes itself with its high selectivity, high sensitivity, and real-time monitoring capabilities [10,11,12,13,14,15,16]. LAS technology achieves precise detection of target gases by analyzing the specific absorption characteristics of gas molecules at specific laser wavelengths. Among these technologies, quartz-enhanced photoacoustic spectroscopy (QEPAS) stands out as a subset of LAS. It utilizes quartz tuning forks (QTFs) to detect variations in acoustic signals, thereby inferring gas concentrations. This technology boasts advantages such as high sensitivity and robust anti-interference capabilities [17,18,19,20,21,22]. However, QEPAS faces practical challenges, particularly when detecting corrosive gases. This challenge arises from the need to expose the QTF to the gas environment, which restricts its range of application. To address this limitation, light-induced thermoelastic spectroscopy (LITES) technology has emerged. In LITES, the laser beam passes through the target gas and directly irradiates the surface of the QTF. By utilizing the thermal effect generated by the gas absorption of laser energy, this method infers the gas concentration [23,24]. This approach not only eliminates the need to directly immerse the QTF in the gas, but also offers significant advantages, including non-contact detection, broadband detection capabilities, and high sensitivity [25,26,27,28,29,30,31,32,33,34,35].

In traditional LITES technology, a multi-pass cell (MPC) is widely used to expand the interaction pathways between the laser and gas, thereby enhancing detection sensitivity. Although the use of an MPC has played a crucial role in boosting detection efficiency, it also presents certain drawbacks, such as its large size and complex optical design, as well as the need for numerous optical components to achieve beam coupling. To tackle the aforementioned challenges, an alternative approach is to employ hollow-core fiber (HCF) for gas sensing applications. In 2020, Hu et al. established a near-infrared all-fiber LITES system by utilizing a hollow-core photonic crystal fiber (HC-PCF) [36]. Nevertheless, the detection performance of this system is constrained by the internal mode interference within the HC-PCF. In 2022, Ma et al. constructed a near-infrared LITES sensing system that was based on a lens set and made use of a hollow-core anti-resonant fiber (HC-ARF) [37]. The distinctive circular structure inherent in the HC-ARF exhibits a remarkable ability to suppress the mode interference noise present in the fiber [38,39,40]. However, the incorporation of lenses brings about several adverse consequences. These include an increase in system losses and size, as well as a decrease in system stability. In order to overcome these limitations, an all-fiber structure in which the HC-ARF serves as both the transmission medium and the gas chamber can be established. The all-fiber structure founded on the HC-ARF offers a multitude of benefits. These advantages encompass lower optical loss, simpler optical alignment procedures, reduced mode interference noise, a smaller sensor size, and cost savings. This innovative design for the transmission medium and gas chamber not only offers an extended absorption pathway but also effectively mitigates some of the drawbacks associated with optical cells, such as bulky size, high cost, and maintenance challenges. The introduction of HC-ARF provides new opportunities for the advancement of LITES technology, promising more efficient, cost-effective, and stable trace gas detection [41,42,43,44,45].

In LITES technology, QTF serves as the core component, influencing the detection performance of sensors. Typically, QTF with a resonant frequency (*f*_0_) of 32.768 kHz is employed as the detection element. However, the energy accumulation time is inversely proportional to the *f*_0_. Therefore, to enhance the energy accumulation time of QTF and thereby convert stronger piezoelectric signals, it is imperative to design a QTF with low frequency [46,47,48,49].

In this paper, an all-fiber LITES sensor based on HC-ARF and self-designed low-frequency QTF was proposed. This all-fiber structure eliminated the need for lens combinations, enhancing the stability and sensitivity of the sensor. HC-ARF served as both the light transmission medium and the gas chamber, while a QTF with a *f*_0_ of 8690.76 Hz was utilized as the detection element to further improve the sensor’s detection capabilities. Acetylene (C_2_H_2_) was employed as the target gas to validate the sensor’s detection performance.

## 2. Experimental Setup

### 2.1. Characteristics of HC-ARF and Self-Designed QTF

In this paper, an all-fiber LITES sensor that makes use of a HC-ARF is presented. The employed HC-ARF is 10 cm in length with a numerical aperture (NA) of 0.02. It is constructed from a single layer of seven non-contacting silica capillaries. The HC-ARF has an outer diameter of 220 µm and features a hollow core with an inscribed inner diameter of 57 µm. The glass wall of the HC-ARF has a thickness of 0.63 µm, which guarantees its functionality within the first anti-resonance band that spans from 1.45 µm to at least 2.4 µm. This wavelength range encompasses all the wavelengths utilized in the experimental study conducted here. Compared with the commonly used HCF in all-fiber structures, the unique circular structure in the HC-ARF demonstrates a remarkable ability to suppress mode interference noise in the fiber. The thickness of the cladding capillary wall plays a crucial role in determining the wavelength order, which can effectively suppress the coupling between the core mode and the cladding mode, thereby confining the majority of the light to propagate within the air core. This characteristic not only significantly mitigates the impact of the substrate material but also substantially reduces the material loss. Furthermore, the laser that excites the vibration of the QTF was directly channeled from the terminal end of the HC-ARF to the surface of the QTF, eliminating the necessity of lens-based focusing. The developed all-fiber structure offers multiple advantages, such as low optical loss, simplified optical alignment, enhanced system stability, compact sensor size, and cost efficiency.

**Figure 1:**
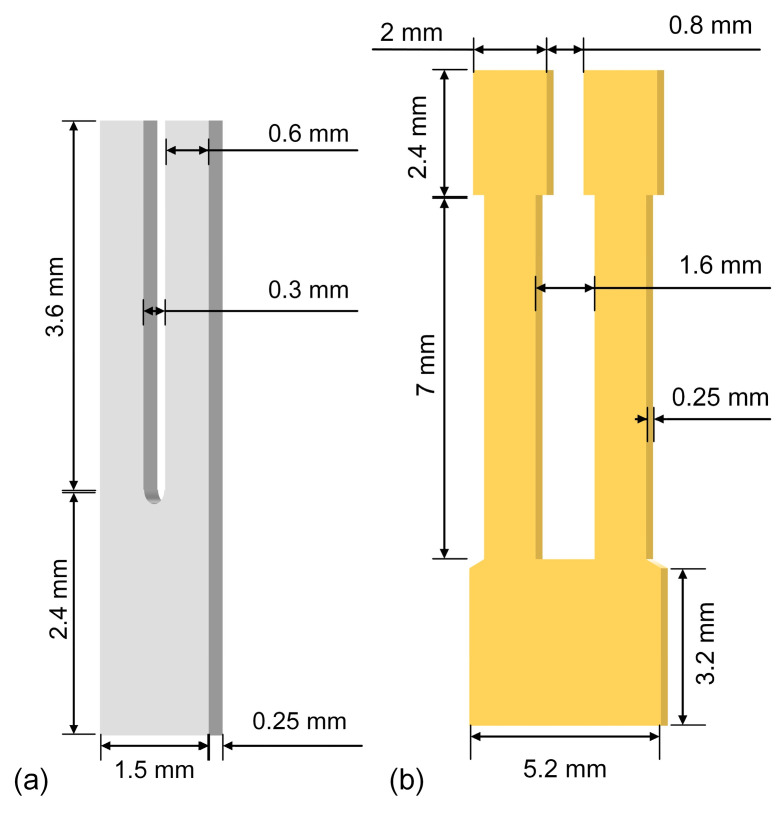
Sizes of two QTFs employed in the experiment. (**a**) The commercial QTF. (**b**) The self-designed rectangular-tip QTF.

In LITES technology, the QTF functions as the core component, exerting an influence on the detection performance of sensors. Conventionally, a QTF with a *f*_0_ of 32.768 kHz is employed as the detection element. However, the energy accumulation time is inversely proportional to *f*_0_. Thus, to augment the energy accumulation time of the QTF and thereby convert stronger piezoelectric signals, it is essential to design a low frequency QTF. Simulation models of a commercial QTF and a self-designed rectangular-tip QTF were established. The commercial QTF has the following dimensions: 3.6 mm (length), 0.6 mm (width), and 0.25 mm (thickness). The remaining size details of the commercial QTF employed in the experiment are illustrated in Figure 1a. The self-designed rectangular-tip QTF has undergone appearance optimization. Finite element analysis was utilized to explore factors of the self-designed rectangular-tip QTF, such as length, finger width, and thickness, with the aim of attaining a stronger average surface charge density, a higher maximum stress distribution, and a lower *f*_0_. The length, width, and thickness of the self-designed rectangular-tip QTF are 9.4 mm, 5.2 mm, and 0.25 mm, respectively. The other dimensional aspects of the self-designed rectangular-tip QTF are depicted in Figure 1b.

**Figure 2:**
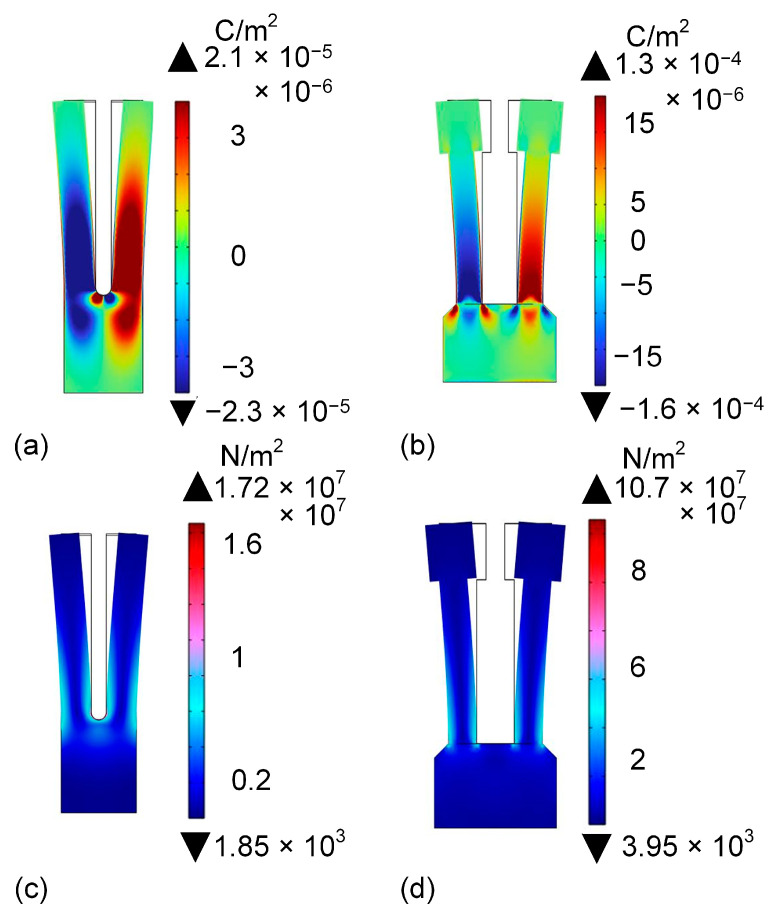
Simulation results of two QTFs. (**a**) Surface charge density of commercial QTF. (**b**) Surface charge density of self-designed rectangular-tip QTF. (**c**) Stress distribution of commercial QTF. (**d**) Stress distribution of self-designed rectangular-tip QTF.

The *f*_0_ of the commercial QTF and the rectangular-tip QTF were determined to be 32,769.42 Hz and 8987.53 Hz, respectively. The surface charge density of the two different QTFs is presented in Figure 2a,b. The stress distribution of the two different QTFs is shown in Figure 2c,d. The average surface charge density and maximum stress distribution of the commercial QTF were 3.28 × 10^−6^ C/m^2^ and 1.72 × 10^7^ N/m^2^, respectively. In comparison with the commercial QTF, the self-designed rectangular-tip QTF demonstrates an increase in average surface charge density and maximum stress distribution. The average surface charge density and maximum stress distribution of the self-designed rectangular-tip QTF were 7.87 × 10^−6^ C/m^2^ and 1.07 × 10^8^ N/m^2^, respectively. The average surface charge density and maximum stress distribution were enhanced by 2.4-fold and 6.22-fold, respectively, relative to the commercial one. The unique rectangular tip of the self-designed rectangular-tip QTF elevates the center of gravity, thereby enabling the QTF to generate more robust piezoelectric signals during vibration.

**Figure 3:**
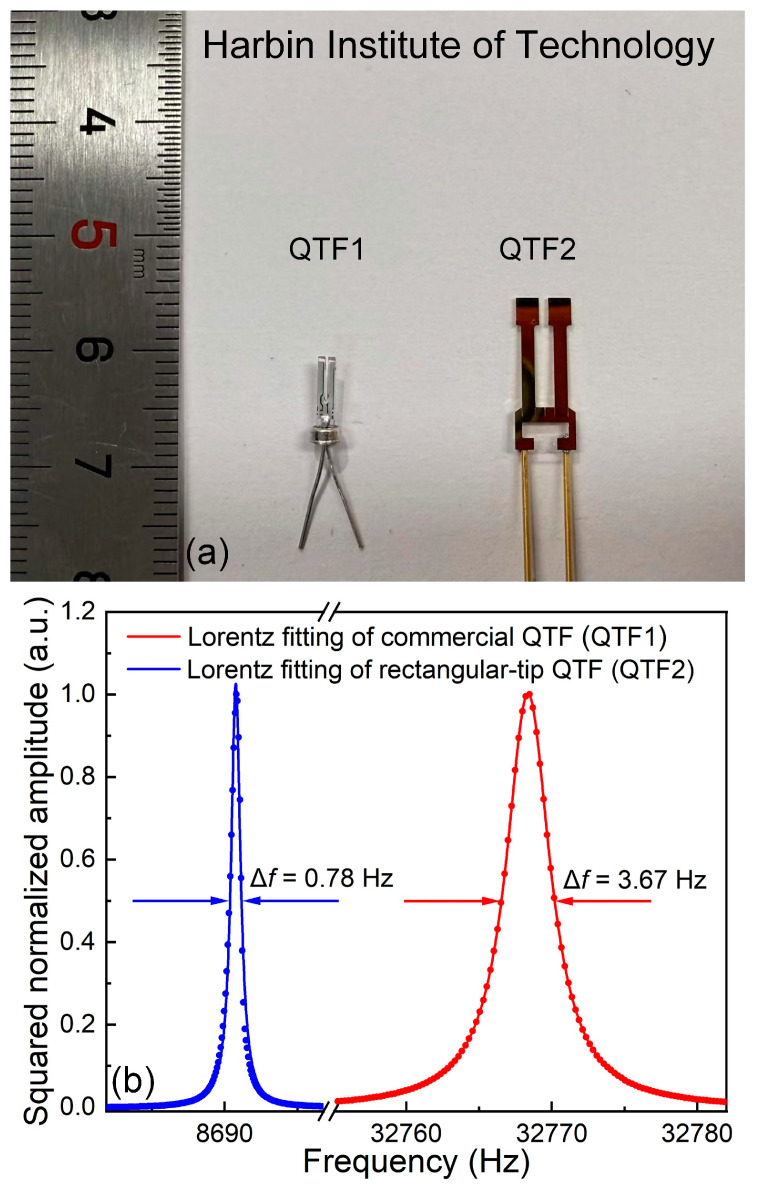
Characteristics of two QTFs. (**a**) Physical images of two QTFs. (**b**) Frequency response curves of commercial QTF (in red) and rectangular-tip QTF (in blue).

The two QTFs utilized in this experiment are depicted in Figure 3a. QTF1 and QTF2 represent a commercial QTF and a self-designed rectangular-tip QTF, respectively. The frequency response curves of the two QTFs are illustrated in Figure 3b, with Lorentz fitting applied to the data. Experimental results indicate that the *f*_0_ of the commercial QTF and the rectangular-tip QTF are 32,768.36 Hz and 8690.76 Hz, respectively, with response bandwidths (Δ*f*) of 3.67 Hz and 0.78 Hz, respectively. According to the formula Q = *f*_0_/Δ*f*, the quality factor (Q) values of the commercial QTF and the rectangular-tip QTF are calculated as 8929 and 11,142, respectively. Compared to the commercial QTF, the rectangular-tip QTF exhibits a *f*_0_ reduction of approximately 73%, which is advantageous for enhancing the sensor’s detection performance. Additionally, the rectangular-tip QTF features gold-plated electrodes, offering better oxidation and corrosion resistance, compared to the silver-plated electrodes used in commercial QTFs.

### 2.2. HC-ARF-Based LITES Sensor Structure

**Figure 4:**
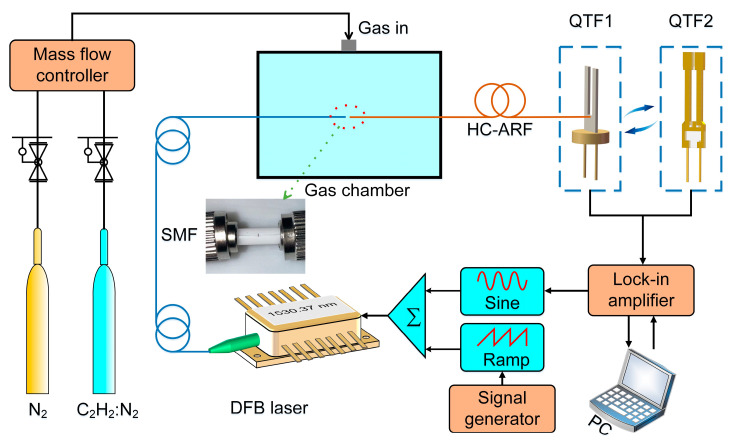
Schematic diagram of all-fiber LITES sensor based on HC-ARF.

The schematic diagram of the all-fiber LITES sensor based on HC-ARF is presented in Figure 4. In the selection process of the target absorption line, the following three principles should be followed. First, the absorption line must have sufficient intensity to obtain a strong enough signal. Second, the selected absorption line should be separated from the spectral lines of background gases to avoid spectral interference. Lastly, the laser used in the experimental conditions must be capable of achieving the required wavelength. Based on these principles, in this experiment, C_2_H_2_, featuring an absorption line at 6534.37 cm^−1^ (1.53 μm), was chosen as the target gas to validate the sensor’s performance. A continuous wave (CW), distributed-feedback (DFB) diode laser was selected as the excitation source. The laser in this wavelength operates in the communication band, offering advantages such as low cost and fiber-coupled output. The signal generator produced a low-frequency sawtooth wave with a scanning period of 50 s to scan the gas absorption line, while the lock-in amplifier generated a high-frequency sine wave to modulate the laser wavelength. These two waves were superimposed and jointly affected the laser. During the experiment, the integration time of the lock-in amplifier was set to 200 ms. The SMF used in the experiment is SMPF0215-APC (Thorlabs) with an NA of 0.14. The laser tail fiber was connected with the SMF and was spatially coupled with the HC-ARF using an optical fiber fixture, and the coupling region was positioned within a 3D-printed gas chamber with a size of 130 × 60 × 35 mm and a thickness of 3 mm, leaving a gap of ~500 μm between the two fibers to facilitate gas entry. Once the laser was coupled to the HC-ARF, it underwent a thorough reaction with the gas and subsequently irradiated directly onto the root of the QTF through the end of the HC-ARF, where the QTF underwent maximum elastic deformation. The electrical signal generated by the QTF was demodulated by the lock-in amplifier to retrieve gas concentration information. Additionally, the experiment was conducted at room temperature. To increase the pressure inside the gas chamber and facilitate the filling of the gas to be measured in the HC-ARF, the designed 3D-printed gas chamber was equipped with only an inlet port, and the end of the HC-ARF served as the outlet port. The air pressure at the inlet was 780 Torr, and the air pressure at the fiber output end was 760 Torr. To adjust the concentration of the test gas in the experiment, two mass flow controllers were used to regulate the flow rates from a bottle of pure nitrogen (N_2_) and a bottle containing a 2% C_2_H_2_:N_2_ standard gas mixture. This all-fiber structure eliminated the need for lens combinations, significantly reducing the system’s size and cost while enhancing its stability.

## 3. Experimental Results and Discussion

**Figure 5:**
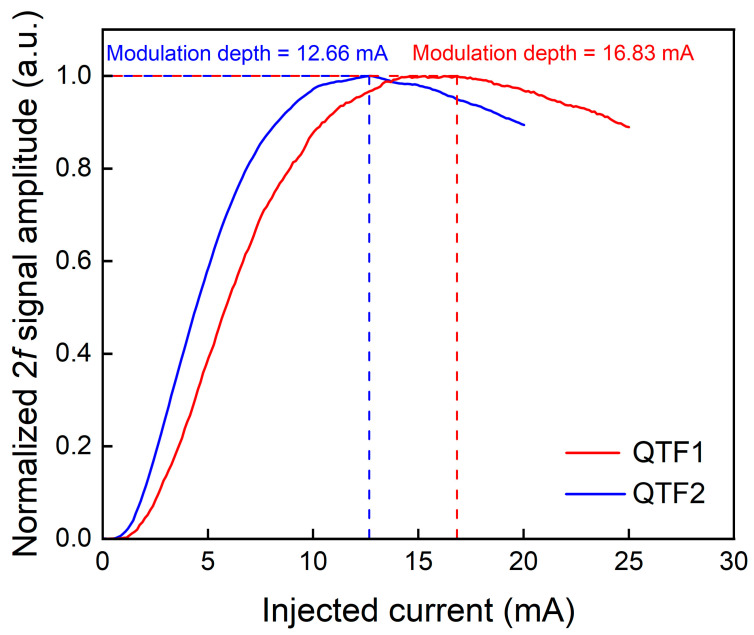
The relationship between current modulation depth and signal amplitude of QTF1 (in red) and QTF2 (in blue).

In the experiment, wavelength modulation spectroscopy (WMS) and the second harmonic (2*f*) demodulation techniques were used to suppress background noise. Modulation depth is an important parameter that affects the signal amplitude of the sensing system. Due to the use of two QTFs with different resonant frequencies, the laser modulation frequencies corresponding to the two QTFs are also different, resulting in different optimal current modulation depths. The relationship between the current modulation depth and signal amplitude of QTF1 and QTF2 is shown in Figure 5. The experimental results indicated that the optimal modulation depths for QTF1 and QTF2 were determined as 16.83 mA and 12.66 mA, respectively. Therefore, in the following experiments these two values are adopted.

**Figure 6:**
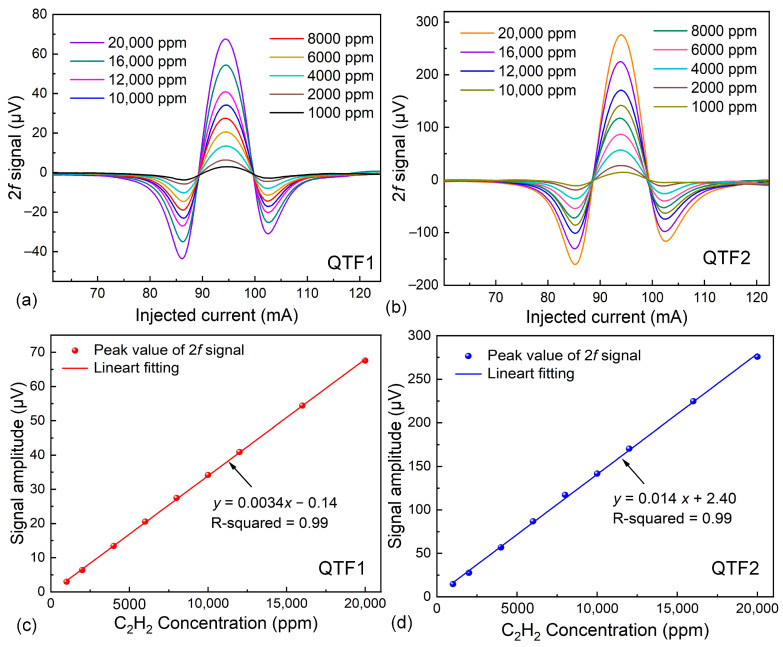
(**a**) When using QTF1, the 2*f* signals corresponding to different concentrations of C_2_H_2_. (**b**) When using QTF2, the 2*f* signals corresponding to different concentrations of C_2_H_2_. (**c**) The relationship between different concentrations of C_2_H_2_ and corresponding 2*f* signal peaks when using QTF1. (**d**) The relationship between different concentrations of C_2_H_2_ and corresponding 2*f* signal peaks when using QTF2.

To verify the detection performance of the sensor, the 2*f* signals were detected by QTF1 and QTF2 at different C_2_H_2_ concentrations. The total flow rate controlled by two mass flow controllers was set to 240 mL/min. Varying concentrations of C_2_H_2_ were generated by adjusting the flow rates of two mass flow controllers. The response speed of the all-fiber LITES system is determined by the filling time of the gas. Under the given experimental conditions, it took approximately 2 min for the gas to completely fill the entire HC-ARF. Figure 6a,b depict the 2*f* signals corresponding to different C_2_H_2_ concentrations when QTF1 and QTF2 serve as detection elements. At a 2% C_2_H_2_ concentration, the peak value of the 2*f* signal measured by QTF1 is 67.55 μV, whereas the peak value measured by QTF2 is 275.79 μV, which is 4.08 times higher than that of QTF1. This superior performance is attributed to QTF2’s excellent energy accumulation time. Figure 6c,d display the peak values of 2*f* signals corresponding to varying C_2_H_2_ concentrations. Through linear fitting, it is evident that both sensors exhibit excellent linear concentration response to C_2_H_2_.

**Figure 7:**
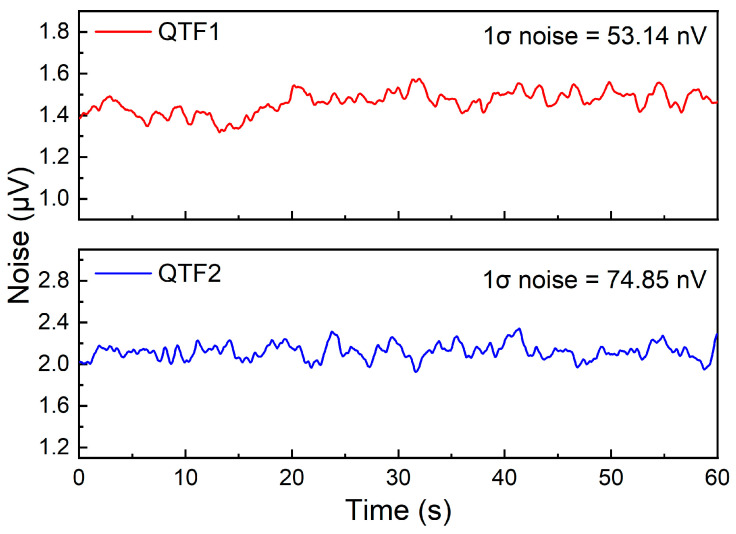
The noise of the all-fiber LITES sensor system when QTF1 and QTF2 are used as detection elements.

The experiment necessitates measuring system noise to ascertain the minimum detection limit (MDL) of the system. By setting the laser output wavelength at the gas absorption peak and introducing pure N_2_ gas, continuous monitoring of signal amplitude is achieved. The system noise results for QTF1 and QTF2 are exhibited in Figure 7. The standard deviations (1σ) for both QTFs are 53.14 nV and 74.85 nV, with signal-to-noise ratios (SNR) of 1271 and 3685, respectively. Consequently, the MDLs for C_2_H_2_ for QTF1 and QTF2 are determined to be 15.74 ppm and 5.43 ppm, respectively. It is evident that the utilization of the rectangular-tip QTF leads to a 2.9-fold improvement in the detection performance. This compelling result serves as strong validation that our self-designed rectangular-tip QTF possesses substantial performance superiority compared to the commercial QTF.

**Figure 8:**
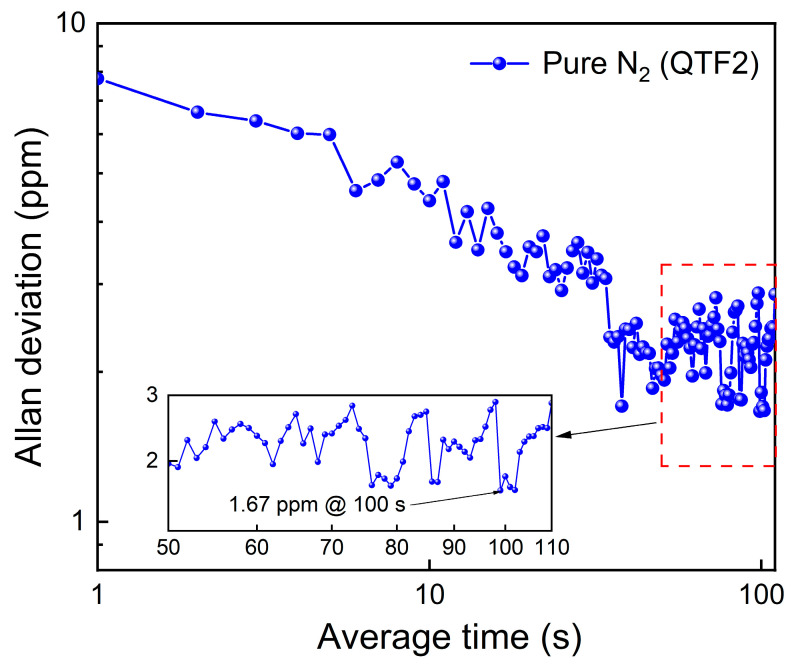
Allan deviation analysis for the all-fiber LITES sensor system.

Figure 8 presents the Allan deviation result of the all-fiber LITES sensor system using QTF2. The result was obtained by setting the laser output wavelength at the absorption peak, introducing pure N_2_ gas, and continuously monitoring the signal for over 2.5 h. Evidently, when the average time is set at 100 s, the MDL of the all-fiber LITES sensor system can be improved to 1.67 ppm, indicating the system’s excellent stability.

## 4. Conclusions

In summary, this article presents an all-fiber LITES sensor based on HC-ARF and arectangular-tip QTF. The HC-ARF was spatially coupled with the laser tail fiber at one end, while the other end was directly guided to the QTF surface, resulting in an all-fiber structure that enhances system stability. The QTF was optimized, and a rectangular-tip QTF with a *f*_0_ of 8690.76 Hz was realized. C_2_H_2_ was selected as the target gas to evaluate the sensor’s detection performance. Experimental results indicate that the MDLs of C_2_H_2_ for commercial QTF and the rectangular-tip QTF are 15.74 ppm and 5.43 ppm, respectively, with the latter showing a 2.9-fold improvement. The Allan deviation analysis shows that the all-fiber LITES sensor based on the rectangular-tip QTF achieves an MDL of 1.67 ppm at an average time of 100 s.

## Data Availability

The data presented in this study are available on request from the corresponding authors.

## References

[1] Liu K., Wang L., Tan T., Wang G., Zhang W., Chen W., Gao X. (2015). Highly sensitive detection of methane by near-infrared laser absorption spectroscopy using a compact dense-pattern multipass cell. Sens. Actuators B Chem..

[2] Wang R., Qiao S., He Y., Ma Y. (2025). Highly sensitive laser spectroscopy sensing based on a novel four-prong quartz tuning fork. Opto-Electron. Adv..

[3] Zifarelli A., Sampaolo A., Patimisco P., Giglio M., Gonzalez M., Wu H., Dong L., Spagnolo V. (2023). Methane and ethane detection from natural gas level down to trace concentrations using a compact mid-IR LITES sensor based on univariate calibration. Photoacoustics.

[4] Wang G., Cui R., Di J., Wang J., Wang Y., Shang Z., Liu X., Tian Q., Wu H., Dong L. (2025). Portable methane sensor system using miniature multi-pass cell for mobile monitoring of natural gas leaks. Sens. Actuators B Chem..

[5] Zifarelli A., Negro G., Mongelli L.A., Sampaolo A., Ranieri E., Dong L., Wu H., Patimisco P., Gonnella G., Spagnolo V. (2024). Effect of gas turbulence in quartz-enhanced photoacoustic spectroscopy: A comprehensive flow field analysis. Photoacoustics.

[6] Mei H., Wang G., Xu Y., He H., Yao J., He S. (2024). Simultaneous measurement of methane, propane and isobutane using a compact mid-infrared photoacoustic spectrophone. Photoacoustics.

[7] Qiao S.D., He Y., Sun H.Y., Patimisco P., Sampaolo A., Spagnolo V., Ma Y.F. (2024). Ultra-highly sensitive dual gases detection based on photoacoustic spectroscopy by exploiting a long-wave, high-power, wide-tunable, single-longitudinal-mode solid-state laser. Light Sci. Appl..

[8] Wang X., Qiu X., Liu M., Liu F., Li M., Xue L., Chen B., Zhang M., Xie P. (2023). Flat soliton microcomb source. Opto-Electron. Sci..

[9] Sun B., Patimisco P., Sampaolo A., Zifarelli A., Spagnolo V., Wu H., Dong L. (2023). Light-induced thermoelastic sensor for ppb-level H_2_S detection in a SF_6_ gas matrices exploiting a mini-multi-pass cell and quartz tuning fork photodetector. Photoacoustics.

[10] Ran S.X., Ni W.J., Yang C.Y., Zhao Z.K., Lin Q.S., He B.Z., Wu R.M., Shum P.P. (2025). Fiber-tip photothermal transducer with gold-coated multi-beam interferometric cavity for high sensitivity gas detection. Appl. Phys. Lett..

[11] Wang Y., Zhang J., Zheng Y., Xu Y., Xu J., Jiao J., Su Y., Lü H.-F., Liang K. (2023). Brillouin scattering spectrum for liquid detection and applications in oceanography. Opto-Electron. Adv..

[12] Shao M., Ji C., Tan J., Du B., Zhao X., Yu J., Man B., Xu K., Zhang C., Li Z. (2023). Ferroelectrically modulate the Fermi level of graphene oxide to enhance SERS response. Opto-Electron. Adv..

[13] Zhang C., Qiao S., He Y., Liu C., Ma Y. (2025). Multi-resonator T-type photoacoustic cell based photoacoustic spectroscopy gas sensor for simultaneous measurement C_2_H_2_, CH_4_ and CO_2_. Sens. Actuators B Chem..

[14] Li T., Zhao P., Wang P., Krishnaiah K.V., Jin W., Zhang A.P. (2024). Miniature optical fiber photoacoustic spectroscopy gas sensor based on a 3D micro-printed planar-spiral spring optomechanical resonator. Photoacoustics.

[15] Han X., Li C., Qi H., Peng W., Chen K. (2025). Carbon black absorption enhanced fiber-optic photoacoustic gas sensing system with ultrahigh sensitivity. Anal. Chem..

[16] Lou C., Li X., Chen H., Yang X., Zhang Y., Yao J.-Q., Liu X. (2021). Polymer-coated quartz tuning fork for enhancing sensitivity of laser-induced thermoelastic spectroscopy. Opt. Express.

[17] Feng C., Shen X., Li B., Liu X., Jing Y., Huang Q., Patimisco P., Spagnolo V., Dong L., Wu H. (2024). Carbon monoxide impurities in hydrogen detected with resonant photoacoustic cell using a mid-IR laser source. Photoacoustics.

[18] Wang L., Lv H., Zhao Y., Wang C., Luo H., Lin H., Xie J., Zhu W., Zhong Y., Liu B. (2024). Sub-ppb level HCN photoacoustic sensor employing dual-tube resonator enhanced clamp-type tuning fork and U-net neural network noise filter. Photoacoustics.

[19] Zhang F.C., Camarero P., Haro-González P., Labrador-Páez L., Jaque D. (2023). Optical trapping of optical nanoparticles: Fundamentals and applications. Opto-Electron. Sci..

[20] Wang J.P., Wu H.P., Liu X.L., Wang G., Wang Y., Feng C.F., Cui R.Y., Dong L. (2024). Cantilever-enhanced dual-comb photoacoustic spectroscopy. Photoacoustics.

[21] S Russo S.D., Pelini J., Garcia I.L., Canino M.C., Roncaglia A., Pastor P.C., Galli I., De Natale P., Borri S., de Cumis M.S. (2024). Dual-tube MEMS-based spectrophone for sub-ppb mid-IR photoacoustic gas detection. Photoacoustics.

[22] Sun H.Y., He Y., Qiao S.D., Liu Y.H., Ma Y.F. (2024). Highly sensitive and real-simultaneous CH_4_/C_2_H_2_ dual-gas LITES sensor based on Lissajous pattern multi-pass cell. Opto-Electron. Sci..

[23] Sun H., Qiao S., He Y., Sun X., Ma Y. (2025). Parts-per-quadrillion level gas molecule detection: CO-LITES sensing. Light Sci. Appl..

[24] Shang Z., Wu H., Wang G., Cui R., Li B., Gong T., Guo G., Qiu X., Li C., Dong L. (2025). Robust and compact light-induced thermoelastic sensor for atmospheric methane detection based on a vacuum-sealed subminiature tuning fork. Photoacoustics.

[25] Wang Z., Nie Q.X., Sun H.J., Wang Q., Borri S., Natale P.D., Ren W. (2024). Cavity-enhanced photoacoustic dual-comb spectroscopy. Light Sci. Appl..

[26] Li A., Wu Y., Wang C., Bao F., Yang Z., Pan S. (2024). An inversely designed integrated spectrometer with reconfigurable performance and ultra-low power consumption. Opto-Electron. Adv..

[27] Cheng Y., Xu Y., Chen T., Mei H., He S. (2025). Differential laser-induced thermoelastic spectroscopy for dual-gas CO_2_/CH_4_ detection. Measurement.

[28] Zhang H., Wang Z., Wang Q., Borri S., Galli I., Sampaolo A., Patimisco P., Spagnolo V.L., De Natale P., Ren W. (2023). Parts-per-billion-level detection of hydrogen sulfide based on doubly resonant photoacoustic spectroscopy with line-locking. Photoacoustics.

[29] Li S., Yuan Y., Shang Z., Yin X., Sampaolo A., Patimisco P., Spagnolo V., Dong L., Wu H. (2023). Ppb-level NH_3_ photoacoustic sensor combining a hammer-shaped tuning fork and a 9.55 µm quantum cascade laser. Photoacoustics.

[30] Golmohamadi H., Keypour R., Mirzazade P. (2021). Multi-objective co-optimization of power and heat in urban areas considering local air pollution. Eng. Sci. Technol. Int. J..

[31] Y Ma Y., Sui X., Song F., Chang Z., Zhang Y., Zheng C., Wang Y., Tittel F.K. (2024). Optical-domain modulation cancellation method for background-suppression and dual-gas detection in light-induced thermo-elastic spectroscopy. Sens. Actuators B Chem..

[32] Zhou T., Wu T., Wu Q., Chen W.D., Wu M.W., Ye C.W., He X.D. (2020). Real-time monitoring of ^13^C-and ^18^O-isotopes of human breath CO_2_ using a mid-infrared hollow waveguide gas sensor. Anal. Chem..

[33] Zhang D., Zhang H., Fan H., Hu M., Wang H., Zhou J., Lv J., Liang J., Wang Q. (2024). Cavity-enhanced light-induced thermoelastic spectroscopy for trace-gas sensing. Opt. Express.

[34] Ma Y., Liu Y., He Y., Qiao S., Sun H. (2025). Design of multipass cell with dense spot patterns and its performance in a light-induced thermoelastic spectroscopy-based methane sensor. Light Adv. Manuf..

[35] Li Z., Chen J., Li L., Zhang J., Yao J. (2023). Exceptional-point-enhanced sensing in an all-fiber bending sensor. Opto-Electron. Adv..

[36] Hu L., Zheng C., Zhang Y., Zheng J., Wang Y., Tittel F.K. (2020). Compact all-fiber light-induced thermoelastic spectroscopy for gas sensing. Opt. Lett..

[37] Ma Y., Feng W., Qiao S., Zhao Z., Gao S., Wang Y. (2022). Hollow-core anti-resonant fiber based light-induced thermoelastic spectroscopy for gas sensing. Opt. Express.

[38] Gomółka G., Pysz D., Buczyński R., Nikodem M. (2024). Wavelength modulation spectroscopy of oxygen inside anti-resonant hollow-core fiber-based gas cell. Opt. Laser Technol..

[39] Bojęś P., Jaworski P., Pokryszka P., Belardi W., Spagnolo V., Krzempek K. (2023). Dual- band light-induced thermoelastic spectroscopy utilizing an antiresonant hollow-core fiber-based gas absorption cell. Appl. Phys. B.

[40] Jiang S., Chen F., Zhao Y., Gao S., Wang Y., Ho H.L., Jin W. (2023). Broadband all-fiber optical phase modulator based on photo-thermal effect in a gas-filled hollow-core fiber. Opto-Electron. Adv..

[41] Liu Z., Dong S., Zhang L., Liu H., Zhou Z., Dong Y., Yang T. (2024). Exhaustive design and statistical analysis of HC-ARFs based on geometric modeling. J. Light. Technol..

[42] Jing Y., Du H., Hua J., Li X., Li J., Li S. (2025). Polarization filter of hollow-core anti-resonant fiber in the 1550 nm band based on SPR effect. Opt. Laser Technol..

[43] Pei S., Nie Q., Liu Z., Cheng M., Yang D., Wang Y., Gao S., Cheng C., Guo D., Yang M. (2024). Atmospheric environment monitoring by antiresonant fiber-enhanced Raman Spectroscopy with sub-ppm sensitivity. IEEE Sens. J..

[44] Z Zhang Z., Zhou M., Wang C., Wang Y., Guo X., Zhou C., Ruan S. (2023). Temperature-immune Fabry-Perot cavity sensor based on an opened hollow-core anti-resonant fiber. Opt. Express.

[45] Li Y., Chen H., Chen Q., Li H., Gao Z. (2023). Surface plasmon resonance induced methane gas sensor in hollow core anti-resonant fiber. Opt. Fiber Technol..

[46] Bi S., Zhang X., Zhang Z., Liu X., Qin L., Shi J., Zhao Y., Wang Z. (2025). A light-induced thermoelastic spectroscopy using surface mounted device quartz tuning fork. Photoacoustics.

[47] Chen X., Qu R., Liu H., Yao L., Xu Z., Hu M., Wang W., Kan R. (2025). MXene-coated quartz tuning fork for sensitive light-induced thermoelastic spectroscopy. Opt. Express.

[48] Erbaş K.C., Erdoğan M., Serdaroğlu D.Ç., Koçum I.C. (2024). A game-changing equation during the etching of tuning forks and its verification through experiments. Measurement.

[49] C Feng C., Zifarelli A., Menduni G., Sampaolo A., Wu H., Dong L., Spagnolo V., Patimisco P. (2024). Low frequency quartz tuning fork as hydrogen sensor. Int. J. Hydrogen Energ..

